# GRHL2 motif is associated with intratumor heterogeneity of cis-regulatory elements in luminal breast cancer

**DOI:** 10.1038/s41523-022-00438-6

**Published:** 2022-06-08

**Authors:** Kohei Kumegawa, Yoko Takahashi, Sumito Saeki, Liying Yang, Tomoyoshi Nakadai, Tomo Osako, Seiichi Mori, Tetsuo Noda, Shinji Ohno, Takayuki Ueno, Reo Maruyama

**Affiliations:** 1 Cancer Cell Diversity Project, NEXT-Ganken Program, Japanese Foundation for Cancer Research, Tokyo, Japan; 2 Breast Surgical Oncology, Breast Oncology Center, Cancer Institute Hospital, Japanese Foundation for Cancer Research, Tokyo, Japan; 3 Project for Cancer Epigenomics, Cancer Institute, Japanese Foundation for Cancer Research, Tokyo, Japan; 4 Division of Pathology, Cancer Institute, Japanese Foundation for Cancer Research, Tokyo, Japan; 5 Project for Development of Innovative Research on Cancer Therapeutics, Cancer Precision Medicine Center, Japanese Foundation for Cancer Research, Tokyo, Japan; 6 Director’s room, Cancer Institute, Japanese Foundation for Cancer Research, Tokyo, Japan; 7 Breast Oncology Center, Cancer Institute Hospital, Japanese Foundation for Cancer Research, Tokyo, Japan

**Keywords:** Breast cancer, Epigenetics, Tumour heterogeneity, Tumour biomarkers, Transcriptional regulatory elements

## Abstract

In breast cancer patients, tumor heterogeneity is associated with prognosis and therapeutic response; however, the epigenetic diversity that exists in primary tumors remains unknown. Using a single-cell sequencing assay for transposase-accessible chromatin (scATAC-seq), we obtained the chromatin accessibility profiles of 12,452 cells from 16 breast cancer patients including 11 luminal, 1 luminal-HER2, 1 HER2^+^, and 3 triple-negative subtypes. Via this profiling process, tumors were classified into cancer cells and the tumor microenvironment, highlighting the heterogeneity of disease-related pathways including estrogen receptor (ER) signaling. Furthermore, the coexistence of cancer cell clusters with different ER binding motif enrichments was identified in a single ER^+^ tumor. In a cluster with reduced ER motif enrichment, we identified GRHL2, a transcription factor, as the most enriched motif, and it cooperated with FOXA1 to initiate endocrine resistance. Coaccessibility analysis revealed that GRHL2 binding elements potentially regulate genes associated with endocrine resistance, metastasis, and poor prognosis in patients that received hormonal therapy. Overall, our study suggests that epigenetic heterogeneity could lead to endocrine resistance and poor prognosis in breast cancer patients and it offers a large-scale resource for further cancer research.

## Introduction

The biological characteristics of cancer vary greatly depending on the patient. In breast cancer, the hormone receptor (HR) and HER2 expression states are currently utilized to decide on clinical management strategies; however, therapeutic outcomes sometimes differ between patients with the same receptor status. For example, most HR-positive breast cancer patients respond well to endocrine therapy, but some cases are refractory to treatment or may experience late recurrence after dormancy^1,2^. Such divergence in the effectiveness of endocrine therapy may be associated with the heterogeneity of estrogen receptor (ER) expression in breast cancer tissue^3^. At present, however, little is known about the differences in cellular properties between ER-expressing and non-expressing cells in a single HR-positive tumor. Thus, as indicated by the example of ER expression, it is necessary to investigate tumors at single-cell resolution to better understand breast cancer heterogeneity and optimize therapeutic options.

The development of techniques by which to measure transcriptomes (scRNA-seq) or multiple proteins (mass cytometry) at single-cell resolution has enabled researchers to determine gene expression landscapes and cellular diversity in breast cancer cells and the tumor microenvironment (TME)^4–7^. However, gene expression reflects not only cell identity but also cell state and cell cycle^8^, complicating precise discrimination between cell lineages. In addition, single-cell expression profiling is often affected by batch effects^9,10^.

The assay for transposase-accessible chromatin using sequencing (ATAC-seq) is an established method used to profile genome-wide chromatin accessibility. Single-cell ATAC-seq (scATAC-seq) is a suitable method for the analysis of complex mixtures of cells from clinical tumor samples because it allows the identification of gene regulatory programs, i.e., enhancers, at the single-cell level^11,12^. In addition, recent progress in scATAC-seq data analysis has led to the minimization of batch effects and enabled estimations of gene amplification, gene expression, *cis*-regulatory element activity, and transcription factor (TF) motif enrichment^13,14^.

In the present study, we demonstrate that scATAC-seq of human breast cancer samples can be used to successfully classify each cell type composing the tumor, i.e., cancer cells, immune cells, and fibroblasts. We also describe breast cancer cell diversity within and across patients. In our in-depth analysis utilizing chromatin accessibility, we determine the intratumor heterogeneity of breast cancer cells with a focus on ER signaling, and we identify distinct cell populations potentially driven by GRHL2 that may contribute to intrinsic resistance to endocrine therapy. Overall, this study describes breast cancer heterogeneity according to multiple features, such as gene activity, regulatory DNA elements, and trans-acting transcription factors; moreover, it provides a valuable resource that will facilitate further studies in the cancer research community.

## Results

### Single-cell chromatin accessibility profiling of human breast cancer tissue

**Fig. 1:**
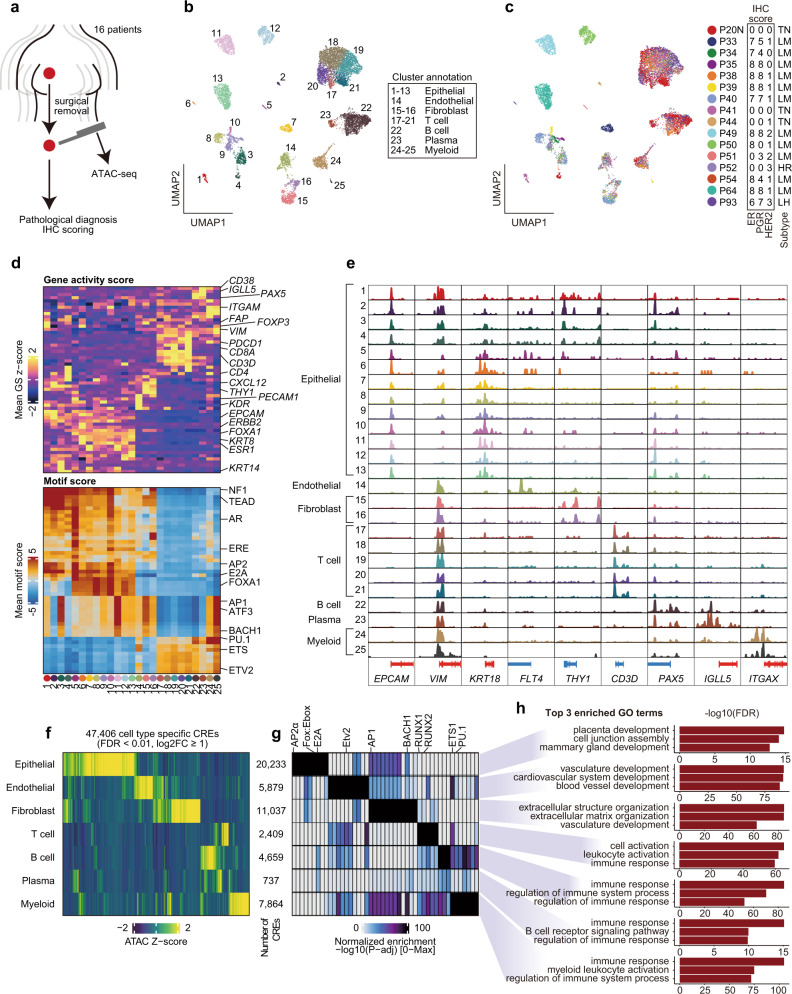
Single-cell chromatin accessibility of human breast cancer tissues. **a** Schema of collecting samples and analysis. **b** Uniform manifold approximation and projection (UMAP) after iterative latent semantic indexing of 12,452 cell scATAC-seq profiles from 16 human breast cancer patients. Each dot represents a single cell colored by its corresponding cluster. Each cluster number is presented on the UMAP. Each cluster annotation is labeled to the right of the UMAP. **c** The same UMAP projection is shown in **a** but with each cell colored by its corresponding patient. Immunohistochemistry scores and breast cancer subtypes are presented on the right of the UMAP. Allred score was used for estrogen and progesterone receptors: ≥3 is considered positive. P20N data were from the primary site diagnosis. Pathological subtypes of each patient are shown as follows: TN triple-negative, LM luminal, HR HER2-positive, and LH luminal-HER2. **d** Marker gene activity score and TF motif scores for each cluster. **e** Aggregated ATAC signal tracks showing the chromatin accessibility peaks of approximate transcription start sites of marker genes for each cluster. Positive-strand genes are colored in red and negative-strand genes are colored in blue. **f** Heatmap of *Z*-score of 47,406 cell-type-specific CREs for each cluster. **g** Heatmap of adjusted *P* value of motif enrichment for each CREs in **f**. **h** Bar plots of top three enriched GO terms calculated by GREAT for each CREs in **f**.

We performed single-cell chromatin accessibility profiling by droplet-based scATAC-seq of 16 prospectively collected samples, including 11 luminal, 1 luminal-HER2, 1 HER2-positive, and 3 triple-negative breast tumors (Table 1). These included 15 primary breast tumors and 1 lymph node metastasis (P20N). Because ATAC-seq requires low inputs, we were able to use breast cancer samples collected by core needle biopsies from surgical specimens (Fig. 1a). We obtained genome-wide chromatin accessibility data from 12,452 cells with sufficient quality, i.e., (i) transcription start site (TSS) score ≥8, (ii) unique nuclear fragments per cell ≥3000, and (iii) an adequate density of fragment size and TSS enrichment profiles (Supplementary Fig. S1a–e).

**Table 1:** Summary of clinical and pathological features for 16 patients.

Patient	Age at surgery	Menopause (age)	Gravidity/Parity	cStage	cTNM	Histological type	ER	PgR	HER2	Subtype Classification	Ki-67 labeling index	Neoadjuvant chemotherapies
P20	54	Post (50)	G3P2	IIIA	cT1cN2aM0	*	0 + 0	0 + 0	0	TNBC	65%	None
P33	35	Pre	G1P1	I	cT1cN0M0	Mucinous	5 + 2	2 + 3	1+	Luminal	25%	None
P34	82	Post (50)	G3P3	I	cT1cN0M0	IDC	5 + 2	3 + 1	0	Luminal	20%	None
P35	45	Pre	G2P1	I	cT1cN0M0	IDC	5 + 3	5 + 3	0	Luminal	3%	None
P38	63	Post (51)	G2P2	I	cT1cN0M0	IDC	5 + 3	5 + 3	1+	Luminal	8%	None
P39	66	Post (52)	G3P2	IIA	cT2N0M0	ILC	5 + 3	5 + 3	1+	Luminal	7%	None
P40	46	Pre	G0P0	I	cT1cN0M0	IDC	5 + 2	5 + 2	1+	Luminal	8%	None
P41	48	Pre	G1P1	I	cT1bN0M0	IDC	0 + 0	0 + 0	0	TNBC	85%	None
P44	60	Post (53)	G2P2	IIA	cT2N0M0	IDC	0 + 0	0 + 0	1+	TNBC	80%	None
P49	73	Post (58)	G0P0	IIA	cT2N0M0	IDC	5 + 3	5 + 3	2 + ,DISH(-)	Luminal	15%	None
P50	61	Post (51)	G1P1	I	cT1cN0M0	IDC	5 + 3	0 + 0	1+	Luminal	10%	None
P51	65	Post (51)	G3P3	I	cT1cN0M0	IDC	0 + 0	2 + 1	2 + ,DISH(-)	Luminal	85%	None
P52	47	Pre	G3P3	IIB	cT2N1M0	**	0 + 0	0 + 0	3+	HER2	65%	CEFx4, DHPx4
P54	67	Post (52)	G0P0	I	cT1bN0M0	ILC	5 + 3	2 + 2	1+	Luminal	5%	None
P64	51	Post (NA)	G3P1	IIA	cT2N0M0	Mucinous	5 + 3	5 + 3	1+	Luminal	20%	None
P93	32	Pre	G0P0	IIB	cT2N1M0	IDC	4 + 2	4 + 3	3+	Luminal-HER2	75%	None

*CEF* cyclophosphamide, epirubicin, and 5FU, *DHP* docetaxel, trastuzumab, and pertuzumab.

*Lymph node metastasis sample.

**Neoadjuvant chemotherapy sample, pPR.

To classify and visualize cell types according to their chromatin accessibility profile, we conducted latent semantic indexing clustering and uniform manifold approximation and projection (UMAP) using a high-quality set of scATAC-seq cells and ArchR^14^; we identified 25 distinct clusters (Fig. 1b, c and Supplementary Table 1). According to the gene activity scores of marker genes, we annotated the clusters as epithelial cells (C1–C13: *EPCAM*^+^ and *KRT*^*+*^), endothelial cells (C14: *KDR*^+^, *ENG*^+^, and *PECAM1*^+^), fibroblasts (C15–C16: *CXCL12*^+^, and *THY1*^+^), T cells (C17–C21: *CD3D*^+^), B cells (C22: *PAX5*^+^), plasma cells (C23: *CD38*^+^ and *IGLL5*^+^), and myeloid cells (C24-25: *ITGAM*^+^ and *ITGAX*^+^) (Fig. 1d, e). The fraction of TME cells was diverse across patients, suggesting that breast cancer TME is highly heterogeneous across patients (Supplementary Fig. S1f, g). Several samples contained a high number of TME cells, especially tumor-infiltrating leukocytes (TILs; T, B, and plasma cells); therefore, we validated the quantity of TILs using pathological assessment based on the International TILS Working Group Scoring^15^ for each sample. The pathological TILs score and proportion of TILs identified via scATAC-seq revealed a significant correlation (Pearson’s correlation coefficient = 0.71, *P* = 0.0032; Supplementary Fig. 1h). We then conducted an unbiased identification of uniquely active genes for each cell type annotated by our knowledge-based approach. Most top-ranked active genes were well-known markers for each cell type: *ENG* for endothelial cells, *COL6A1* for fibroblasts, *BCL11B* for T cells, *TNFRSF13C* for B cells, and *IGLL5* for plasma cells (Supplementary Fig. S1i, j). We also observed the consistency of motif enrichment patterns with cell annotation such as ERE, FOXA1, androgen receptor (AR), and TEAD motifs in the epithelial clusters and ETS family motifs in the immune cell clusters (Fig. 1d).

We next identified cell type-specific *cis*-regulatory elements (CREs). By performing peak calling in ArchR based on the creation of pseudo-bulk replicates, we detected 166,233 reproducible peaks (Supplementary Fig. S1k–m). Approximately 76.8% of peaks were distal CREs, highlighting the importance of distal elements for cellular identity (Supplementary Fig. S1m). Only half of the peaks overlapped between our peaks and a set of peaks identified via bulk ATAC-seq for 74 samples in The Cancer Genome Atlas Breast Invasive Carcinoma (TCGA-BRCA) cohort^16^; this result emphasizes the divergent activity of CREs across breast cancer patients and it extended the set of CREs identified in this study (Supplementary Fig. S1n). Finally, we determined 47,406 cell-type-specific differentially accessible regulatory elements (FDR <0.01; log2FC ≥ 1; Fig. 1f and Supplementary Table 2). Motif analysis for each set of cell type-specific elements revealed the enrichment of lineage-specific TF motifs: RUNX1/2 and the ETS family were enriched for immune cells, whereas the FOX family, Ebox, and E2A motifs were enriched only for epithelial cell clusters (Fig. 1g and Supplementary Table 3). GREAT^17^ GO enrichment analysis identified cell type-specific GO term enrichment for each set of regulatory elements: mammary gland development for epithelial cells, blood vessel development for endothelial cells, extracellular matrix organization for fibroblasts, and immune response for immune cells (Fig. 1h). These results support the consistency of our gene score-based cell type assignment and offer cell-type-specific CREs in breast cancer TMEs.

### Chromatin accessibility landscape of breast cancer cells

**Fig. 2:**
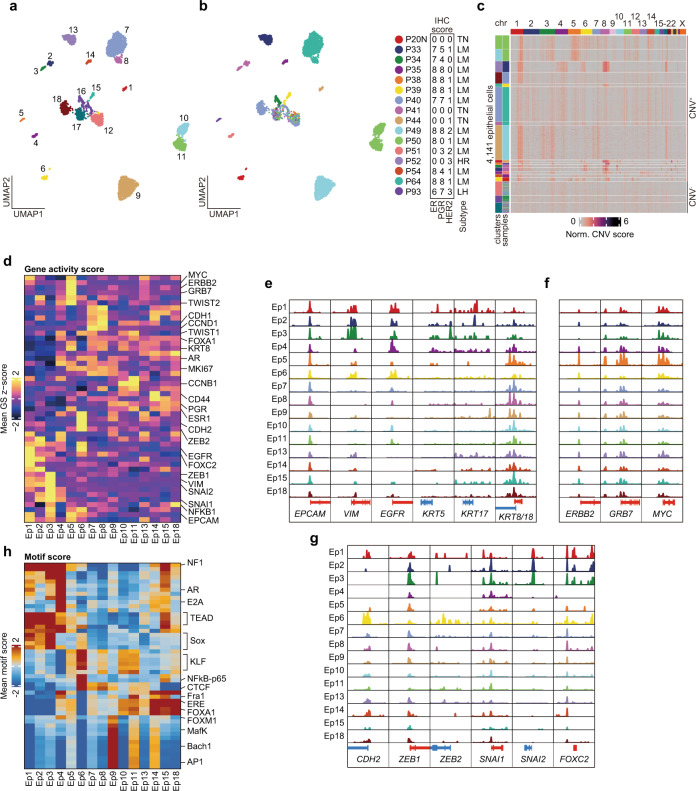
Chromatin landscape of breast cancer cells. **a**, **b** UMAP of subclustering of 4141 epithelial cells, colored according to corresponding epithelial clusters in **a** and corresponding patients in **b**. In **a**, cluster number is shown on the UMAP. Sample details shown to the right of **b** are identical to those shown in Fig. 1b. **c** Inferred copy number variations of 4141 epithelial cells from scATAC-seq data. The log2(fold change) to GC-matched background was calculated and normalized by the same scores for TME cells. Clusters and samples of each cells were represented on the left side of the heatmap. **d** Heatmap of *Z*-score of marker gene activity score. **e**–**g** Aggregated ATAC signal tracks showing chromatin accessibility peaks of approximate transcription start sites of luminal or TNBC markers in **e**, HER2 associated genes in **f**, and epithelial to mesenchymal transition markers in **g** for each cancer cluster. **h** Most variable 50 TF motif scores for each cancer cluster.

To describe the epigenetic heterogeneity of breast cancer cells, we performed subclustering of the 4141 epithelial cells and identified 18 clusters (Ep1–18) (Fig. 2a, b and Supplementary Table 3). Only three clusters (Ep12, 16, and 17) were derived from multiple samples, although most clusters were derived from single patients. This observation was consistent with previously reported scRNA-seq studies indicating that cancer cells were prone to be clustered as per patient samples but that noncancer cells were clustered by cell types regardless of the patient of origin^18–21^.

To determine malignant epithelial clusters, we applied an estimation method for copy number alterations from scATAC-seq;^11^ specifically, we identified the clusters with or without inferred copy number variations (CNVs). After calculating the CNV score for epithelial and TME clusters (Supplementary Fig. 2a), we normalized epithelial CNV scores by TME scores as background (See Method; Fig. 2c). Consistent with a previous report^22^, chromosomes 1q and 8q were frequently amplified in CNV^+^ clusters (Ep1–11, 13–15, and 18). These genomic regions in CNV^−^ clusters (Ep12, 16, and 17) were less frequently amplified, similar to that in immune and stromal cell clusters (Fig. 2c and Supplementary Fig. 2a). CNV^−^ clusters comprised cells from multiple samples across subtypes, suggesting that they were juxtatumoral epithelial cells. Few epithelial cells were obtained from HER2^+^ patient P52, and these were distributed only to CNV^−^ clusters, implying that sampling difficulties could be attributed to tumor size reduction by neoadjuvant therapy in P52 (Table 1 and Supplementary Table 3). Although the numbers of epithelial cells derived from two luminal samples (P38 and P54) were very low, a sufficient number of cells (>50 cells) was obtained from 13 samples, indicating that the sampling method, i.e., core needle biopsy of surgical specimens, was viable, to a certain extent, for the analysis of primary tumors via single-cell ATAC-seq.

After excluding CNV^−^ clusters as juxtatumoral cells, we evaluated gene activity and motif enrichment scores in each cancer cell. TNBC-derived clusters (Ep1–3 and 6) and luminal-derived clusters (Ep4, 5, 7–11, 13–15, and 18) typically had different gene activity patterns. Basal cell markers (*VIM*, *EGFR*, and *KRT5/17*) were highly activated in TNBC-derived clusters, whereas luminal cell markers (*KRT8/18* and *FOXA1*) were active in luminal-derived clusters (Fig. 2d–e and Supplementary Fig. 2b). Additionally, differential motif analysis revealed a heterogeneous pattern of motif enrichment for each cluster, e.g., Sox family in Ep3, AP-1 and TEAD motifs in Ep9, and FOXA1 in Ep14 and Ep18 (Supplementary Table 5). Ep5 from a luminal-HER2-type tumor showed a luminal-specific pattern of gene activity (*KRT8/18*) as well as greater *ERBB2*, *GRB7*, and *MYC* activities, which are often coamplified in breast cancer^23,24^ (Fig. 2f). We also observed an increased activity of epithelial-mesenchymal transition markers (*ZEB1, ZEB2, SNAI1, SNAI2*, and *FOXC2*) as well as the high enrichment of metastasis-associated TFs (TEAD and Sox) in TNBC-derived clusters (Fig. 2g, h). In the lymph node-derived cluster Ep6, *CDH2* and *ZEB2* were strongly activated, which may have been associated with metastatic features (Fig. 2g and Supplementary Fig. 2b). Ep4, which was derived from a P51 tumor pathologically assigned to the luminal type because of their weakly positive progesterone receptor (PR) without ER expression (Allred scores: ER, 0; PR, 3; Fig. 2b), had low *VIM* activity but the high activity of EGFR and relatively low activity of luminal keratins (Fig. 2d, e and Supplementary Fig. 2b). Additionally, AR gene activity and motif enrichment were high in Ep4 (Fig. 2h and Supplementary Fig. 2c), which suggests that Ep4 was a “luminal-AR”-like tumor which was an AR-driven TNBC with luminal-like expression profiles^25^. Immunohistochemistry revealed that AR and FOXA1 expression was high in the P51 sample, confirming the results of chromatin accessibility profiling (Supplementary Fig. 2d). Then, we clearly described the heterogeneity of breast cancer cells across patients, which was consistent with the pathological assessment. This suggests that single-cell epigenome profiling can reveal not only the heterogeneity of regulatory element activity but also cancer cell diversity, including copy number alterations, gene regulatory programs, and TF activities.

### Chromatin accessibility is consistent with the transcriptome in primary breast cancer and TMEs

To validate chromatin accessibility-based single-cell profiling for primary breast cancer, we reanalyzed recently published large-scale scRNA-seq data^26^. Of the 184,116 transcriptome profiles obtained from 38 primary breast or metastatic lymph node samples of 32 patients, we identified 32 clusters using Seurat^27^, which enabled clear classification of breast cancer and TME cells (Supplementary Fig. 3a–b). By subclustering TME cells, we identified the following: T (TME1, 4, and 9; *CD3*^+^), B, and plasma (TME5, 6, 11, 12, and 15; *CD19*^+^) cells; macrophages and myeloid (TME2, 7, 16, and 17; *ITGAM*^+^); dendritic cells (TME14; Toll-like receptor signaling genes); endothelial (TME8; *PECAM*^+^), fibroblast (TME3; *FAP*^+^), pericyte (TME10; *MCAM*^+^, *PDGFRB*^+^, *NOTCH3*^+^), and contaminated (TME13; *KRT8/18* + ) breast cells (Supplementary Fig. 1c–e and Supplementary Table 6). Based on the observations from a shared expression program for TMEs among patients (Fig. 1c and Supplementary Fig. 3d; refs. ^17–20^), we integrated the chromatin profiling with the transcriptome in TME cells by comparing the gene score matrix from our scATAC-seq with the gene expression matrix from the scRNA-seq; accordingly, we identified a similar same cell type annotation (Supplementary Fig. 3f). Thus, scATAC-seq-based estimation for gene expression (i.e., gene score) was consistent with actual gene expression levels when determining TME properties.

**Fig. 3:**
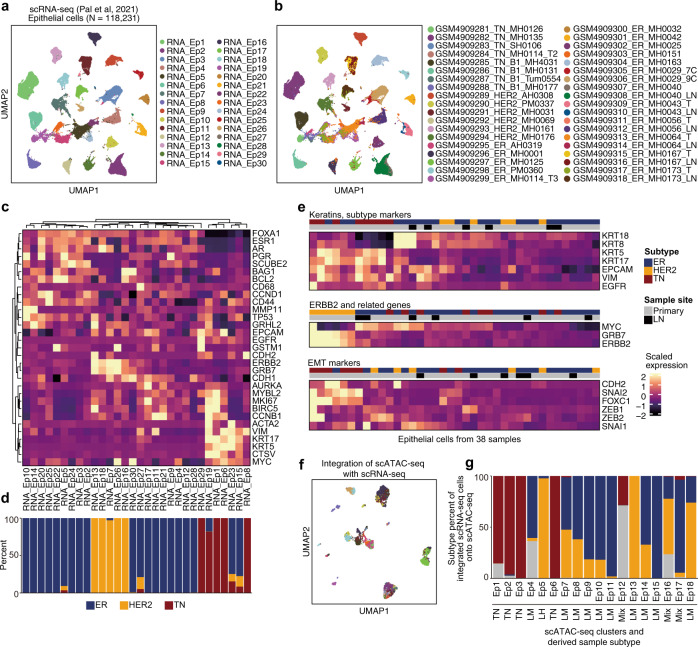
Integrative analysis of previously reported scRNA-seq results and our scATAC-seq data. **a**, **b** UMAP of subclustering of the epithelial cell transcriptome reported in a previous scRNA-seq study (Pal et al., 2021), colored according to the corresponding epithelial clusters in **a** and corresponding samples in **b**. **c** Heatmap of scaled expression of marker genes. **d** Breast cancer subtype composition of each epithelial cluster, according to the scRNA-seq. **e** Heatmap of scaled expression of genes corresponding to the results in Fig. 2e–g. Unlike panel in **c**, the cells are grouped by sample and average scaled expression is presented. **f** UMAP visualization of 4,141 epithelial chromatin profiles, colored according to the linked scRNA-seq clusters presented in panel **a**. Based on gene activity scores, scATAC-seq cells were linked to scRNA-seq exhibiting a similar expression pattern. **g** Subtype composition of scRNA-seq cells associated with each scATAC-seq cluster.

Subsequently, we performed subclustering of 118,231 epithelial cells, identifying 30 clusters (Fig. 3a). Consistent with our scATAC-seq profiling, epithelial cells tended to be clustered per patient (Fig. 3b and Supplementary Table 7); expression of basal cell markers (*VIM*, *EGFR*, and *KRT5/17*) was high in TNBC-derived clusters, whereas luminal cell markers (*KRT8/18* and *FOXA1*) were highly expressed in luminal-derived clusters (Fig. 3c, d). We also examined the gene expression levels of keratins, ERBB2-related genes, and EMT markers in the epithelial cells of each sample. We found that basal keratins (*KRT5/17*), *EGFR*, and *VIM* were highly expressed in TNBC samples, whereas ERBB2 and GRB7 expression were commonly high in HER2^+^ and some luminal tumor samples; MYC was coupregulated in some tumors, and EMT markers were relatively highly expressed in TNBC samples compared with their expression in other subtypes (Fig. 3e). In addition to TME cell analysis, we performed cross-platform integration between scATAC-seq and scRNA-seq for epithelial cells (Fig. 3f). Although the linkage between cells partially reflected breast cancer subtypes (Fig. 3g), there was no one-to-one correspondence between patients, indicating that the gene expression and gene regulatory program in cancer cells have strong intertumor heterogeneity. Taken together, these results suggest that chromatin accessibility is consistent with but does not have exactly the same layer of cellular heterogeneity, as the transcriptome in primary breast cancer.

### ER motif enrichment was heterogeneous but ER target genes were commonly activated in luminal tumors

**Fig. 4:**
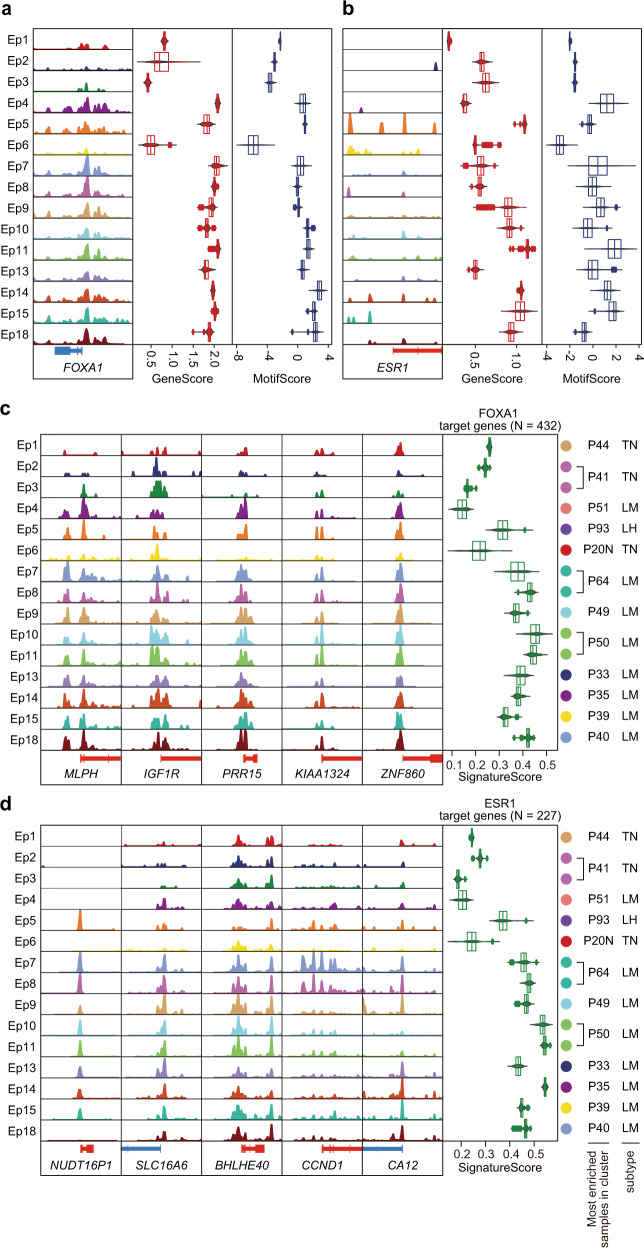
Gene activity signatures of FOXA1 and ER targets. **a**, **b** Aggregated ATAC signal tracks around TSS (left), gene activity scores (center), and motif enrichment scores (right) of FOXA1 in **a** and ESR1 in **b** for each cancer cluster. **c**, **d** Aggregated ATAC signal tracks around TSSs of the represented targets (left) and signature scores (right) of genes targeted by FOXA1 in **c**, ER in **d** for each cancer cluster.

FOXA1 and ER are luminal-lineage TFs associated with breast cancer proliferation, progression, and drug resistance^28–30^. Consistently, *FOXA1* activity and motif enrichment were high in luminal-derived clusters but low in TNBC-derived clusters (Fig. 4a and Supplementary Fig. 4a, c). However, *ER* activity and motif enrichment showed divergent patterns within luminal-derived clusters (Fig. 4b and Supplementary Fig. 4b), which were less correlated (Supplementary Fig. 4c). To explore the heterogeneity of ER binding CREs across patients, we integrated previously reported ER cistrome data^31^ with the epithelial CREs. Our ER motif-containing peaks had 77 overlapping CREs with 2750 previously identified ER-bound sites in normal breast samples (seven overlapping CREs), tumor and normal samples (19 overlapping CREs), and tumor samples (51 overlapping CREs) (Supplementary Fig. 5a). We observed various activities of ER-associated CREs across epithelial clusters (Supplementary Fig. 5b), emphasizing that the accessibility of ER binding elements is highly heterogeneous, even in luminal breast cancers.

To investigate whether the heterogeneity of FOXA1 and ER motif activity influences downstream target transcriptional regulation, we next evaluated the activities of their target genes using a signature score, i.e., an overall activity (inferred expression) measure of each TF target genes calculated via VISION^32^. To calculate the signature score, we used a gene list of breast cancer-specific FOXA1 or ER targets with positive expression correlations of each TF and its target genes as well as a regulatory potential of ≥0.5 in Cistrome Cancer^33^. We obtained 432 genes as FOXA1 targets and 227 genes as ER targets with 115 overlaps (Supplementary Fig. 6a).

Consistent with motif enrichment scores, FOXA1 target signature scores were high in luminal-derived clusters but low in TNBC-derived clusters (Fig. 4c). Although ER motif enrichment was heterogeneous across luminal-derived clusters (Fig. 4b), ER target signature scores were commonly high in luminal-derived clusters but low in TNBC-derived clusters (Fig. 4d). Because FOXA1 and ER targets were partially overlapped, we examined the gene activity scores of mutually exclusive and common targets of FOXA1 and ER (Supplementary Fig. 6b). High activity genes were not biased in the common gene set and the signature scores of each gene set were high in luminal-derived clusters and low in TNBC-clusters, suggesting that the common targets of FOXA1 and ER do not affect the signature scores of each target (Supplementary Fig. 6b, c). We also examined the association between the ER IHC score and ER motif enrichment or ER target signature score; signature scores increased in accordance with ER IHC scores, although the ER motif score was variable even in samples with high IHC scores (Supplementary Fig. 7).

These results suggest that ER-mediated transcription is maintained but gene regulatory elements regulated by ER are heterogeneously activated or inactivated across luminal patients. We speculated that the reprogramming of the ER-mediated gene regulatory program might have occurred in some cases of luminal breast cancer without altering the ER downstream transcriptional output; epigenome could be an essential factor for preexisting heterogeneity in luminal breast cancer.

### GRHL2 emerges as a key TF in decreased ER motif enrichment and targets CREs potentially regulating genes leading to endocrine resistance and poor outcome

**Fig. 5:**
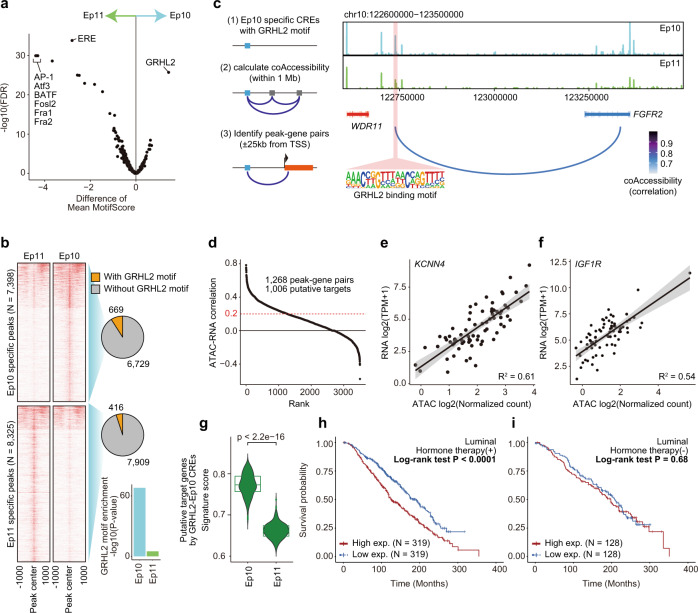
GRHL2 was associated with less ER dependency leading to the intratumor heterogeneity of a single luminal tumor. **a** Volcano plot representing differential motif enrichment between Ep10 and Ep11 from a single luminal tumor. **b** Heatmap of aggregated ATAC-seq signal in a 2 kb window, divided according to a significantly higher signal in Ep11 or in Ep10. Pie charts show the numbers of Ep10- or Ep11-specific peaks with or without GRHL2-binding motifs. The bar plot represents hypergeometric tests of GRHL2 motif enrichment in each CRE set. **c** Schema for identifying peak-gene associations (left), and genome accessibility track of the *FGFR2* region with peak coaccessibility (right). The red band indicates the Ep10-specific element with GRHL2 motif potentially interacting *FGFR2* coding region. **d** Pearson’s correlation between TCGA-BRCA ATAC-seq normalized counts and RNA-seq transcripts per million of each peak-gene pair. **e**, **f** RNA-seq gene expression of *KCNN4* in **e** or *IGF1R* in **f** and normalized ATAC-seq signal of paired peaks in 74 TCGA-BRCA samples. Each dot represents each patient. **g** Signature scores of putative target genes by Ep10-specific CREs with GRHL2 motifs in Ep10 (*N* = 281) and Ep11 (*N* = 327). Wilcoxon rank-sum test was used for significant analysis (*p* < 2.2e-16). **h**, **i** Kaplan–Meier analysis of overall survival of the METABRIC ER^+^HER2^−^ patient cohort (*n* = 1,355) with (*n* = 968, **h**) or without (*n* = 387, **i**) hormone therapy stratified by putative GRHL2 target genes; top 33% versus the bottom 33%, mean of *Z*-score of expression.

To explore the intratumor epigenome heterogeneity of luminal breast cancer, we assessed the luminal tumors P50 and P64 containing two distinct clusters. P50 and P64 had two major clusters (Ep10 and Ep11) as well as major (Ep7) and minor (Ep8) clusters, respectively (Fig. 2a, b and Supplementary Table 4). Differential motif analysis revealed that the ER motif differed most significantly between Ep10 and Ep11 (Fig. 4a and Supplementary Table 8) and that several motifs, including ETS, Elk1, and Ptf1a, were differentially enriched between Ep7 and Ep8 (Supplementary Fig. 8a and Supplementary Table 9). This suggested that the epigenetic divergence between Ep10 and Ep11 was based on the intratumor heterogeneity of the ER-mediated gene regulatory program. Following this, we focused on the P50-derived clusters to determine epigenome diversity associated with ER signaling. TF footprinting analysis also showed that the flanking accessibility of ER motif in Ep10 was decreased compared with that of the ER motif in Ep11. (Supplementary Fig. 8b), suggesting that the subpopulation of cancer cells with the decreased accessibility of ER binding motifs exists within a single luminal tumor. By contrast, Ep10 had significant enrichment of the GRHL2 motif, implying that GRHL2 is associated with reduced ER motif enrichment and expanding intratumor heterogeneity (Fig. 5a and Supplementary Table 8).

To identify GRHL2-binding elements specific to Ep10, we next performed a differential analysis of the accessible regions between Ep10 and Ep11. We identified 7398 Ep10-specific CREs and 8325 Ep11-specific CREs (log2FC > 1; *P* < 0.01), and GRHL2-binding motifs were significantly enriched in Ep10-specific CREs (*P* = 10^−68^, hypergeometric test); 669 (9.04%) of Ep10-specific CREs had GRHL2-binding motifs (Fig. 5b and Supplementary Tables 10, 11). To investigate gene regulatory programs by GRHL2-binding elements in Ep10, we calculated peak coaccessibility and determined genes that are potentially regulated by these 669 elements; we identified 3917 significant peak-gene associations (within ±25 kb around TSSs, correlation >0.6; Fig. 5c). As our peak-gene associations were based on chromatin accessibility data alone, we also confirmed the association between peak signal and gene expression using the patient-matched profiling of chromatin accessibility (ATAC-seq) and transcriptomes (RNA-seq) in the TCGA database^16^ (i.e., matched profiling for 74 patients with breast cancer). Finally, 1006 genes (1268 peak-gene pairs) were identified as potential targets by the GRHL2 motif-containing Ep10-specific CREs (ATAC–RNA correlation >0.2; Fig. 5d and Supplementary Table 12). In these genes, we found protumorigenic genes, including *KCNN4* (*R* = 0.78; Fig. 5e), inducing cell proliferation in breast cancer^34^, and *IGF1R* (*R* = 0.74; Fig. 5f), promoting ER^+^ breast cancer metastasis and endocrine resistance by modulating multiple kinase activities^35–37^, suggesting that the gene regulatory elements are possibly associated with cellular programs for cancer progression. The signature score of putative target genes was significantly high in Ep10 than in Ep11 (Fig. 5g). In the METABRIC database^38,39^, overexpression of putative GRHL2 target genes was significantly associated with poor prognosis in luminal patients who received endocrine therapy; however, associations were not detected in luminal patients that did not receive endocrine therapy (Fig. 5h, i). Thus, high-level expression of these genes could lead to poor outcomes after endocrine therapy.

Taken together, these data suggest that specific CREs were reprogrammed by the master regulator’s shift from ER to GRHL2 within a single luminal tumor, which led to the establishment of tumor subpopulations with potential for intrinsic endocrine resistance and ultimately poor outcomes.

## Discussion

In this study, we describe the epigenetic landscape of the cancer cells and TME in human primary breast cancer. Our integrative framework utilized multiple analytical tools to not only indicate intertumor and intratumor heterogeneity but also provide lists of marker genes and regulatory DNA elements in each breast cancer or TME cell cluster. These sets of features should be useful resources for further studies, such as those including integrative analysis with GWAS data, bulk ATAC-seq data, and other single-cell omics data.

We identified two cancer cell groups with different epigenetic states in a single luminal tumor. Although one had the typical features of a luminal type, the other had distinct CREs with the unique enrichment of TF motifs, especially GRHL2. Overexpression of potential genes targeted by GRHL2-binding CREs, containing metastasis- or endocrine resistance-related genes, was associated with poor prognosis 5 years after diagnosis. GRHL2 has been reported as a TF that reprograms ER signaling during ER^+^ breast cancer tumorigenesis^31^ and also cooperates with FOXA1 to establish endocrine therapy resistance^40^. A recent study revealed that FOXA1 cistrome reprogramming occurred in the development of neuroendocrine prostate cancer after endocrine treatment of prostate adenocarcinoma^41^. According to these reports and our results, we anticipated that although Ep10 has luminal features at the transcriptome level, a subset of FOXA1 changed its partners, i.e., from ER to GRHL2, which reprogrammed cistrome to a less ER-dependent state. This process, in turn, might have caused the intrinsic resistance to endocrine therapy.

This study has several limitations. First, sampling bias and the collection of a low number of cells, which were caused by our sampling procedure, i.e., core needle biopsy. We confirmed that the proportion of TILs in the scATAC-seq profile was strongly correlated with the TILs score in a pathological assessment (Supplementary Fig. 1h); we were able to evaluate the intertumor and intratumor epigenome heterogeneity of epithelial cells from 13 samples, indicating that our sampling method enabled assessment of breast cancer heterogeneity despite relatively low throughput. However, the number of cells per sample varied, and three samples did not have sufficient epithelial cells to enable interpretation (Supplementary Table 4). These limitations make it difficult to (i) compare cell proportions and clinical information, e.g., the effects of a high number of exhausted T cells on patient outcomes and (ii) uncover the interaction between cancer cells and TME cells. To overcome these issues, more input cells, i.e., mass tumor samples must be analyzed or the procedures used in the scATAC-seq experiment must be improved. Second, our findings are based on the analysis of only 16 patient samples, and those on GRHL2 are from a single patient (P50). The low number of patient samples potentially reduces the robustness of our findings. Therefore, extensive research is warranted before we can generalize our results. Studies should include more patient samples and functional analysis. Third, although we analyzed primary tumors to reflect real clinical settings, we did not determine how the epigenetic heterogeneity described above was able to emerge. It will be necessary to observe epigenetic changes over the time course of disease development using several approaches including sampling tumor cells before and after treatment by core needle biopsy and establishing (then testing) patient-derived organoids.

In summary, we demonstrated large-scale single-cell chromatin accessibility profiling of human primary breast cancer. We also provided a data-rich resource, which includes cell type-specific gene sets, CREs, and coaccessibility between regulatory elements in human breast cancer or the TME. By highlighting cistrome heterogeneity, our results help to explain the diversity of therapeutic responses or late recurrence rate in ER-positive breast cancer patients. Finally, we postulate that reprogramming of chromatin accessibility might be a hallmark of breast cancer in a similar manner to genetic alterations or transcriptome changes.

## Methods

### Clinical specimens

Breast cancer specimens were obtained by core needle biopsy of surgically removed tumors. Specimens were dissociated into single cells using a MACS Tumor Dissociation Kit and a gentle MACS dissociator (Miltenyi Biotec) according to the manufacturer’s instructions. All participants gave written informed consent before the collection of specimens. The protocol was approved by the institutional ethical committee of Cancer Institute Hospital, Japanese Foundation for Cancer Research (No. 2018-1168).

### ATAC-seq library preparation

Single-cell ATAC-seq libraries were prepared using a SureCell ATAC-Seq Library Prep Kit (Bio-Rad) and a SureCell ddSEQ Index Kit (Bio-Rad) according to the manufacturer’s instructions. Libraries were loaded at 1.5 pM on a NextSeq 550 (Illumina) and sequencing was performed using the following read protocol: Read 1: 118 cycles; i7 index read: 8 cycles; and Read 2: 40 cycles. FASTQ files were processed using the ATAC-Seq Analysis Toolkit (Bio-Rad) to generate debarcoded and aligned read data.

### Pathological assessment

Unstained formalin-fixed paraffin-embedded tissue sections (4-μm thick) were used for hematoxylin-and-eosin (H&E) staining and immunostaining. The antibodies used in this study are listed as follows: ER (SP1, ready to use; Ventana), PgR (1E2, ready to use; Ventana), HER2 (4B5, ready to use; Ventana), AR (AR441, 1/200; Dako), and FOXA1 (EPR10881-14, 1/1000; Abcam). To assess the proportion of TILs in samples, International TILs Working Group^15^ scoring was used.

### scATAC-seq analysis—ArchR

We used ArchR v.1.0.2 for scATAC-seq analysis^14,26^. All analyses were performed with the hg19 genome assembly using ArchR’s “addArchRGenome(“hg19”)” function. We assessed ATAC-seq quality using by enrichment of ATAC-seq accessibility at tran^14^ scription start sites. ArchR’s “createArrowFiles()” function calculated TSS enrichment score and unique nuclear fragments. We filtered the cells using cut-off of TSS enrichment score of eight and 3000 unique fragments per cell to exclude low-quality cells. To filter out doublets, ArchR’s “addDoubletScores()” with “k = 10, knnMethod = “UMAP”, LSIMethod =1” parameters was used. Quality plots were made by “plotGroups()”, “plotFragmentSizes()”, and “plotTSSEnrichment()”.

ArchR’s “addIterativeLSI()” function with genome-wide 500-bp tile matrix was used for calculating iterative LSI information. We clustered the cells by ArchR’s “addClusters()” with Seurat’s “FindClusters()” and default parameters. To run uniform manifold approximation and projection (UMAP), we used ArchR’s “addUMAP()” function with 40 nearest neighbors.

We visualized gene activity scores in the UMAP overlay by ArchR’s “plotEmbedding()” function. We also obtained a gene score matrix using “getMatrixFromProject()” for getting pre-imputed matrix, “imputeMatrix()” for matrix imputation with MAGIC, and log2(Imputed gene score + 1) for normalization.

For calling peaks on scATAC-seq binary data, pseudo-bulk replicates were made using ArchR’s “addGroupCoverages()” function. Then, MACS2 v2.2.7.1 was called by ArchR’s “addReproduciblePeakSet()” function with parameter of “shift = −40, extsize = 80, --nomodel --nolambda”. The “addPeakMatrix()” was used for adding the merged peak set to ArchR project. We obtained cluster-specific peaks using ArchR’s “getMarkerFeatures()” and “getMarkers()” functions (FDR <0.01 and log2FC ≥1, Wilcoxon rank-sum test). For motif enrichment analysis of these regions, “peakAnnoEnrichment()” and “plotEnrichHeatmap()” were used. For subclustering of epithelial cells, we conducted peak call again to obtain cancer cell-specific peaks.

We measured TF activities by two computational approaches in ArchR: ChromVAR deviation scores and TF footprinting. For ChromVAR analysis, the raw insertion counts for all peaks were used as input. HOMER motif annotations were added by ArchR’s “addMotifAnnotations()” function. We computed the GC bias-corrected motif deviation scores using ArchR’s “addDeviationsMatrix()”. We also obtained the motif score matrix as same as the gene score matrix described above. For TF footprint analysis, we first obtained the positions of HOMER motifs using “getPositions()” function. We then computed footprints of the motifs of interest by “getFootprints()”. To correct Tn5 bias and visualization, ArchR’s “plotFootprints()” function with ‘normMethod = “Subtract”’ was used.

We obtained a peak coaccessibility profile using ArchR’s “addCoAccessibility()” with “maxDist = 1e + 06”, meaning the maximum window size for detecting coaccessibility. We used the coaccessibility information with correlation cut-off of 0.6 and 1-bp resolution.

### scATAC-seq analysis—copy number estimation

We inferred DNA copy number amplification from scATAC-seq data using a method as described in Satpathy et al., 2019 (“08_Run_scCNV_v2.R” script from https://github.com/GreenleafLab/10x-scATAC-2019). We first constructed the 10 kb genome windows using “makeWindows(genome = BSgenome.Hsapiens.UCSC.hg19, blacklist = blacklist)” with a hg19 blacklist bed file from ENCODE portal (ENCFF001TDO.bed). We next obtained CNA profiles using “scCNA()” function with the parameters “neighbors = 100, LFC = 1.5, FDR = 0.1, remove = c(“chrM”, “chrY”)”. This script computes the mean log2(fold change) of ATAC-seq read counts (CNV score) for each window against the GC-matched 100 nearest neighbors to estimate whether an amplification exists. To visualize cancer-specific CNV scores, we subtracted mean scores of TME cells from scores of each cancer cell as “Normalized CNV score”.

### scRNA-seq analysis—Seurat and ArchR

scRNA-seq data by Pal et al., 2020 was downloaded from GEO Accession Viewer (GSE161529). We used Seurat v4.0.5 for scRNA-seq analysis^27^. Each data were read by the Seurat function Read10x(), and merged int a single Seurat object. The data was filtered to remove cells with fewer 500 unique genes per cell and over 20% of mitochondrion RNAs. We processed the data with Seurat’s standard pipeline following steps: (1) NormalizedData() was run using “LogNormalize” method with scale.factor of 10,000, (2) Highly variable features were identified by FindVariableFeatures() using “vst” method and 2,000 features, (3) scaling the data and computing principal components were performed by ScaleData() and RunPCA(), (4) Clustering the cells was conducted by FindNeighbors() function with 1–50 dimensions and FindClusters() function with a resolution of 0.2, (5) the cells were projected onto UMAP embedding space by RunUMAP() with 1–50 dimensions. In subclustering of TME and epithelial cells, the same parameters were used. To integrate scRNA-seq with scATAC-seq, ArchR’s addGeneIntegrationMatrix() function was used. The RNA epithelial clusters were classified into each subtype derived cluster with over 80% cells from a single subtype. The clusters with over 80% cells from a single subtype sample were determined as “mixed clusters”.

### scATAC-seq analysis—VISION

To calculate gene signature score, the R package VISION^32^, a signature-based analytical tool for scRNA-seq was used. We applied the log2 normalized gene score matrix as input to VISION with annotated gene sets in publicly available transcription factor target gene sets in Cistrome Cancer^33^. The functions of “Vision()” for making a vision object, “analysis()” for a signature-based analysis, “getSignatureScores()” for extracting signature scores were used.

### scATAC-seq analysis—Homer

We used HOMER^42^ v.4.10 for identifying differential peaks between Ep10 and Ep11. To obtain differential peaks, we first used “makeTagDirectory” for creating tag directories from each pseudo-bulk bam files. We next merged ArchR’s reproducible peak sets of Ep8 and Ep9 after conversion from GRanges objects to bed files. The “getDifferentialPeaks” was used with “-size given -F 2.0 -P 0.01” options.

### TCGA data analysis—ATAC-seq

We downloaded TCGA chromatin accessibility profiles from National Cancer Institute Genomic Data Commons websites via browser (https://gdc.cancer.gov/about-data/publications/ATACseq-AWG). BRCA specific normalized counts matrix and called peaks were used in this study (BRCA_log2norm.txt and BRCA_peakCalls.txt). The peak position is annotated by hg38 assembly, so we lifted over hg38 to hg19 using UCSC Genome Browser Lift Genome Annotations (https://genome.ucsc.edu/cgi-bin/hgLiftOver). We found overlap peaks between our scATAC-seq peaks and TCGA-BRCA peaks lifted over hg38 to hg19 using “findOverlaps()” in the GenomicAlignments package.

### TCGA data analysis—RNA-seq

TCGA-BRCA RNA-seq data was downloaded using the R package “TCGAbiolinks”^43^. For the generation of an expression matrix, the HTSeq counts data for each primary tumor was used. After selecting samples matching to the TCGA case IDs with matched ATAC-seq, we computed the exon lengths for each gene following steps: (i) reading a downloaded exon annotation file “gencode.v38.annotation.gtf” using “makeTxDbFromGFF()” in GenomicFeatures package, (ii) obtaining an exon list using “exonBy(by = ‘gene’)”, and (iii) summing the non-overlapping exons by “lapply(x, function(x){sum(width(reduce(x))”). We excluded all genes mapping to “chrM”, and converted length normalized the RNA-seq data to transcripts per million (TPM).

### TCGA data analysis—Integration for ATAC-seq and RNA-seq

PAM50-based subtype profile was obtained using “PanCancerAtlas_subtype()” function in TCGABiolinks. We calculated pearson’s correlation between patient-matched RNA-seq TPM and ATAC-seq normalized counts for each peak-gene pairs using “cor.test(method = “pearson”)” function.

### METABRIC data analysis

METABRIC^38,39^ microarray expression data (*Z*-score to all samples) was downloaded using the R package “cBioPortalData”^44^. We selected the luminal patients with positive “ER_STATUS” and negative “HER2_STATUS”, the TNBC patients with negative of “ER_STATUS”, “PR_STATUS”, and “HER2_STATUS”. Hormone therapy status was obtained “HORMONE_THERAPY” column. “OS_STATUS” and “OS_MONTHS” were used for survival analysis. For Kaplan–Meier analysis, we stratified patients by average expression of common genes (*n* = 813) between GRHL2 target genes we identified (*n* = 1006) and METABRIC expression genes (*n* = 24,360) to top 33% and bottom 33%, and then used “survfit()” and “survdiff()” in the “survival” package.

### Reporting Summary

Further information on research design is available in the Nature Research Reporting Summary linked to this article.

## Supplementary information

Supplementary Information resupplied

Reporting Summary

Supplementary data

## Data Availability

Processed scATAC-seq data have been deposited at GEO (Accession ID: GSE198639).

## Code availability

All analysis code is available from the lead contact upon request. Data analysis was performed by R version 4.0.5, ArchR version 1.0.2, MACS2 version 2.2.7.1, Seurat version 4.0.5, and Homer version 4.10. The default variables or parameters were used unless otherwise mentioned in the Method section.
