# Supplementary material for: GRHL2 motif is associated with intratumor heterogeneity of cis-regulatory elements in luminal breast cancer
Source: NPJ Breast Cancer. 2022 Jun 8;8:70. doi: 10.1038/s41523-022-00438-6 (PMC9177858; doi:10.1038/s41523-022-00438-6)
Supplement: Supplementary file 1 — Supplementary Information resupplied [file 41523_2022_438_MOESM1_ESM.pdf]

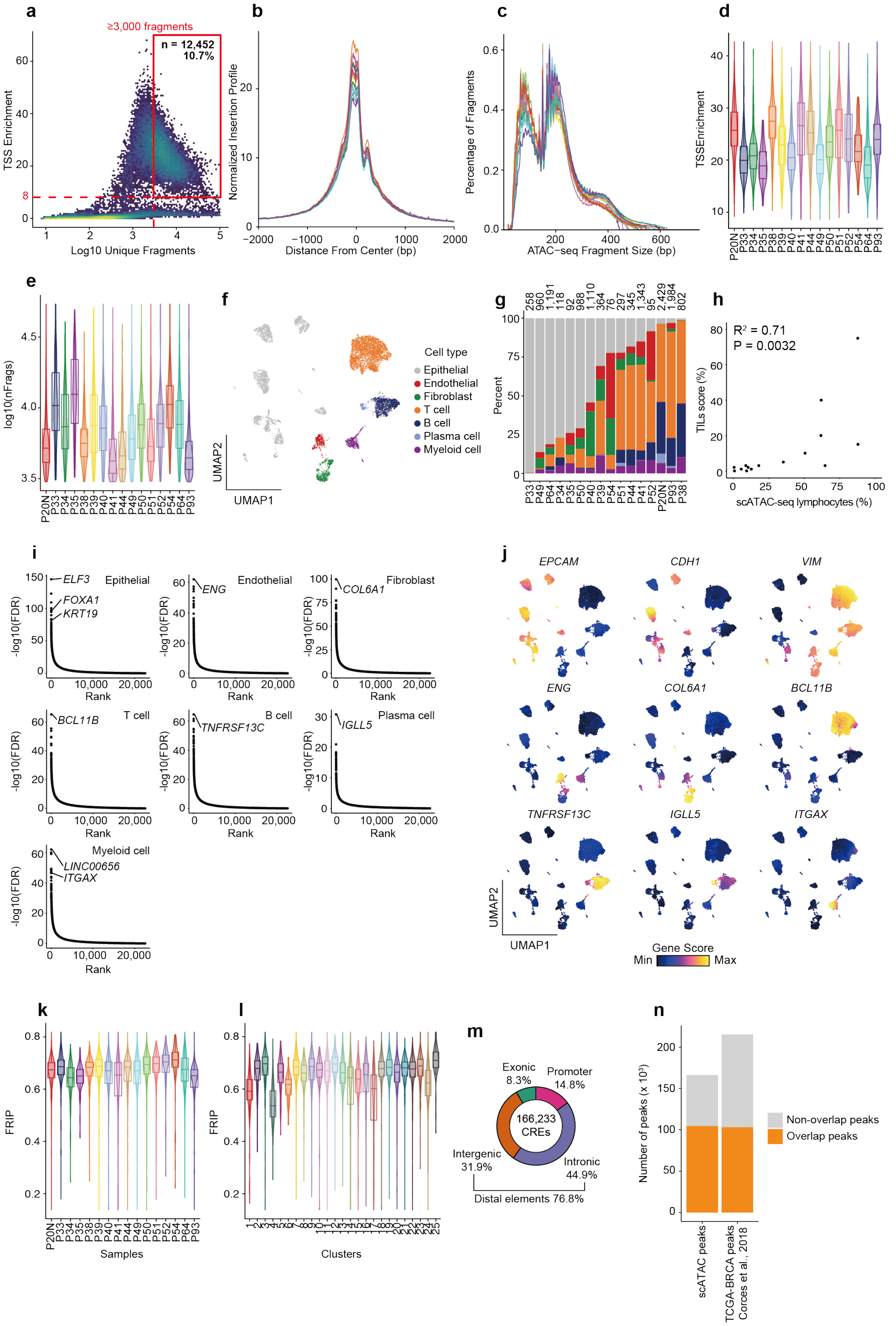


**Supplementary Figure 1. scATAC-seq QC, cell type annotation, and peak profiling.**

a Scatter plots of TSS enrichment score and number of fragments. Each dot represents each cell. Cells in a red box were defined as high quality and further analyzed. b-e scATAC-seq quality control. TSS enrichment in b and d, insertion profiles in c, number of fragments in e for each breast cancer sample. f UMAP plot colored by the annotated cell types. g Percentage of cells of each cluster in each individual samples. Top number means total cell number of each sample. h Correlation plot between percent of tumor infiltrating leukocytes (TILs) identified by scATAC-seq and pathological TILs score. i Differential activated genes in each cell type. Wilcoxon test was used for significant analysis. j UMAP overlay for gene activity scores of marker or significant genes for each cell type. k, l Fraction of reads in peaks for each sample in k, each cluster in l. m Genomic annotation for each peak identified by ArchR. n Bar plot showing the overlap of bulk ATAC-seq of TCGA-BRCA and our scATAC-seq peaks. TCGA ATAC-seq peaks annotated in hg38 lifted over to in hg19. Overlap is considered as any overlapping bases.

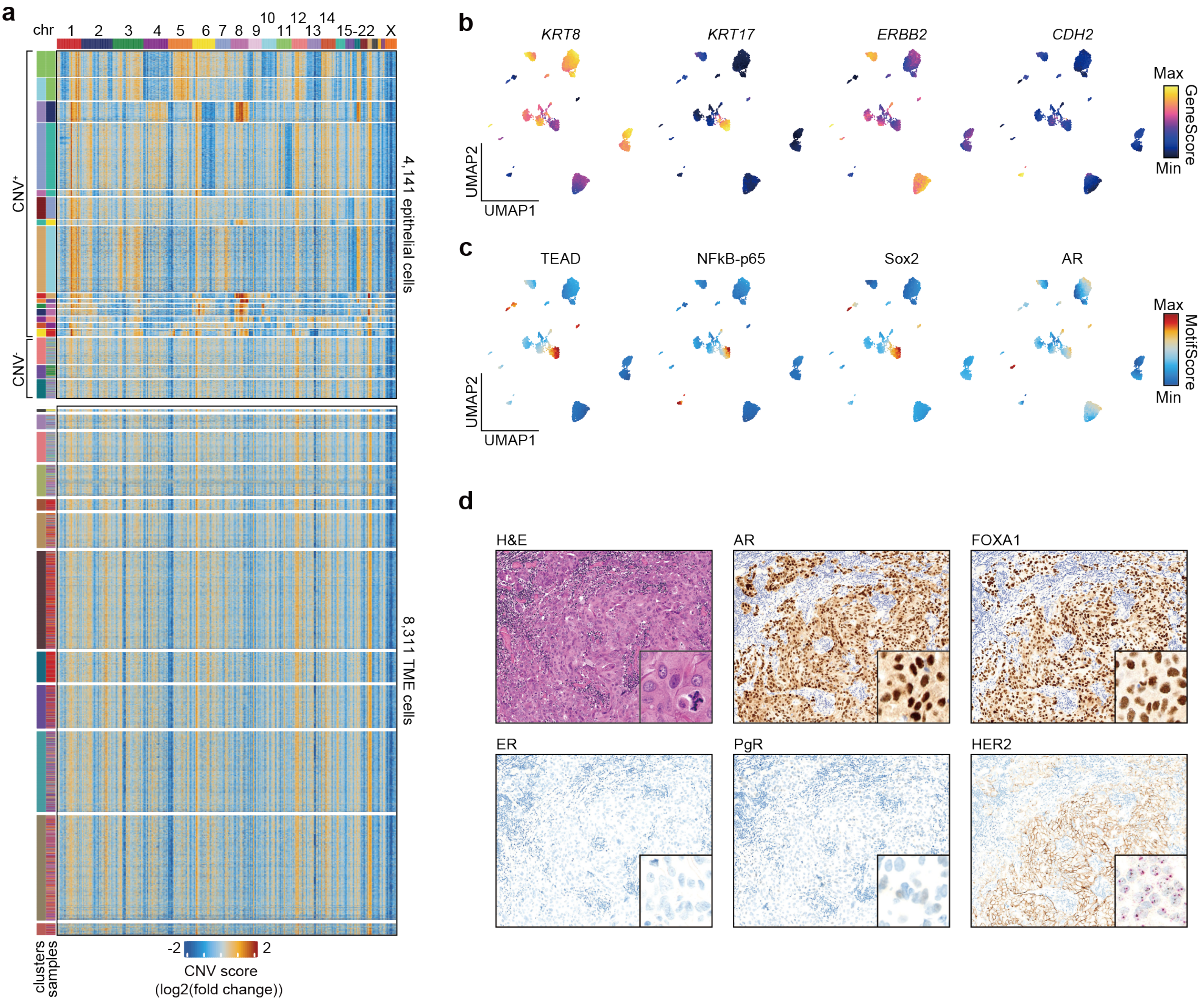


**Supplementary Figure 2. Epigenome profiling for epithelial cells**

a Inferred copy number variations from scATAC-seq data. The log2(fold change) to GC-matched background was calculated for epithelial cells (top) and TME cells (bottom). Clusters and samples of each cell were represented on the left side of the heatmap. b, c UMAP overlay of defined gene activities in b, and motif enrichments in c. d Hematoxylin & Eosin staining and immunohistochemical staining of AR, FOXA1, ER, PGR, and HER2 for luminal-AR like tumor P51 sample. Inset of HER2 staining represents the dual color in situ hybridization (DISH).

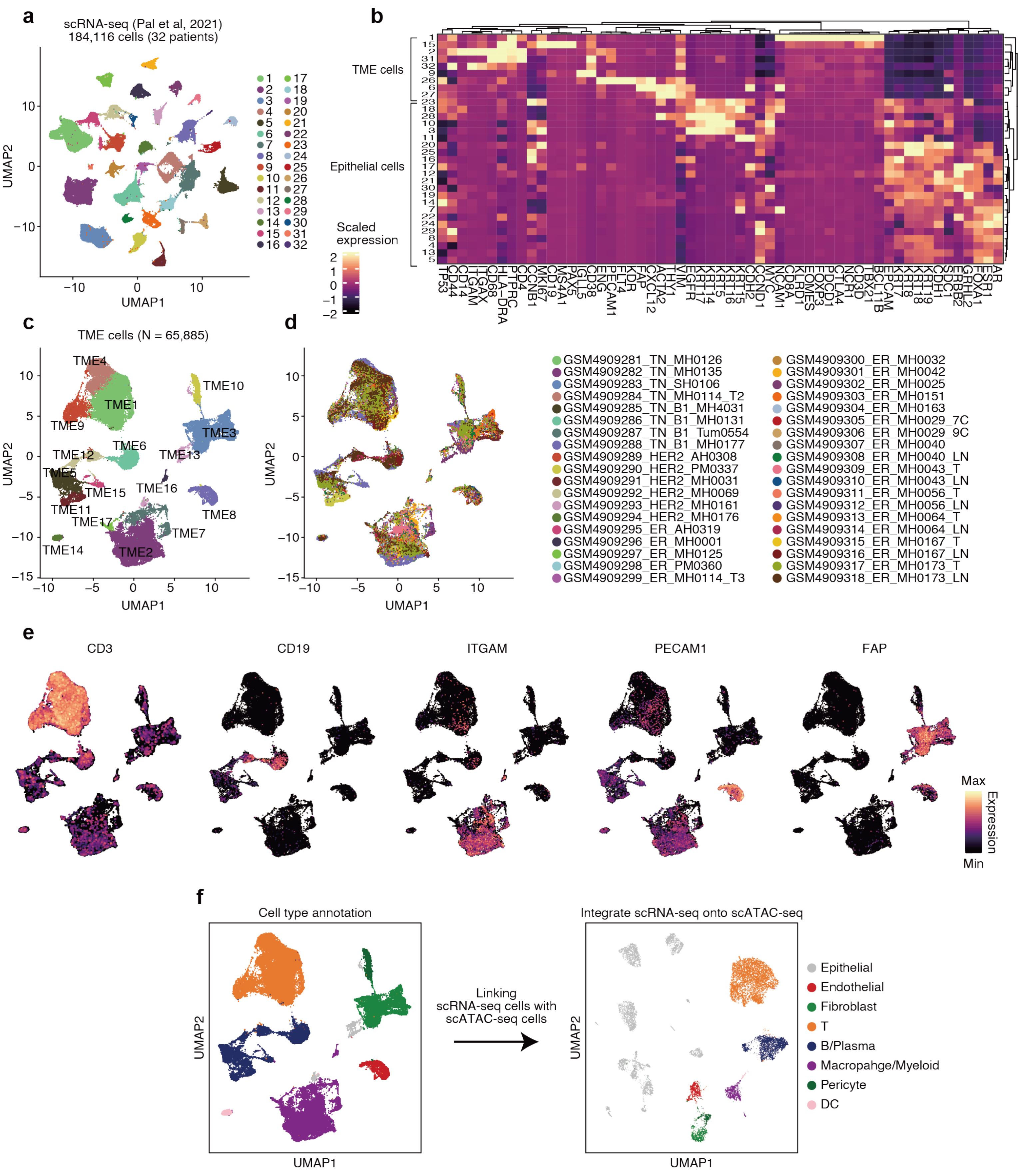


**Supplementary Figure 3. Reanalyzing scRNA-seq for primary breast cancer.**

a UMAP embedding of 184,116 transcriptomic profiling, colored by clusters. b Heatmap of scaled expression of cell type specific marker genes in each cluster. c, d UMAP of subclustering of 65,885 TME cells, colored by TME clusters in c, colored by its corresponding samples in d. e UMAP overlay for gene expression of TME markers. f gene expression based cell type annotation of scRNA-seq cells (left) and the projected annotation onto our scATAC-seq cells (right).

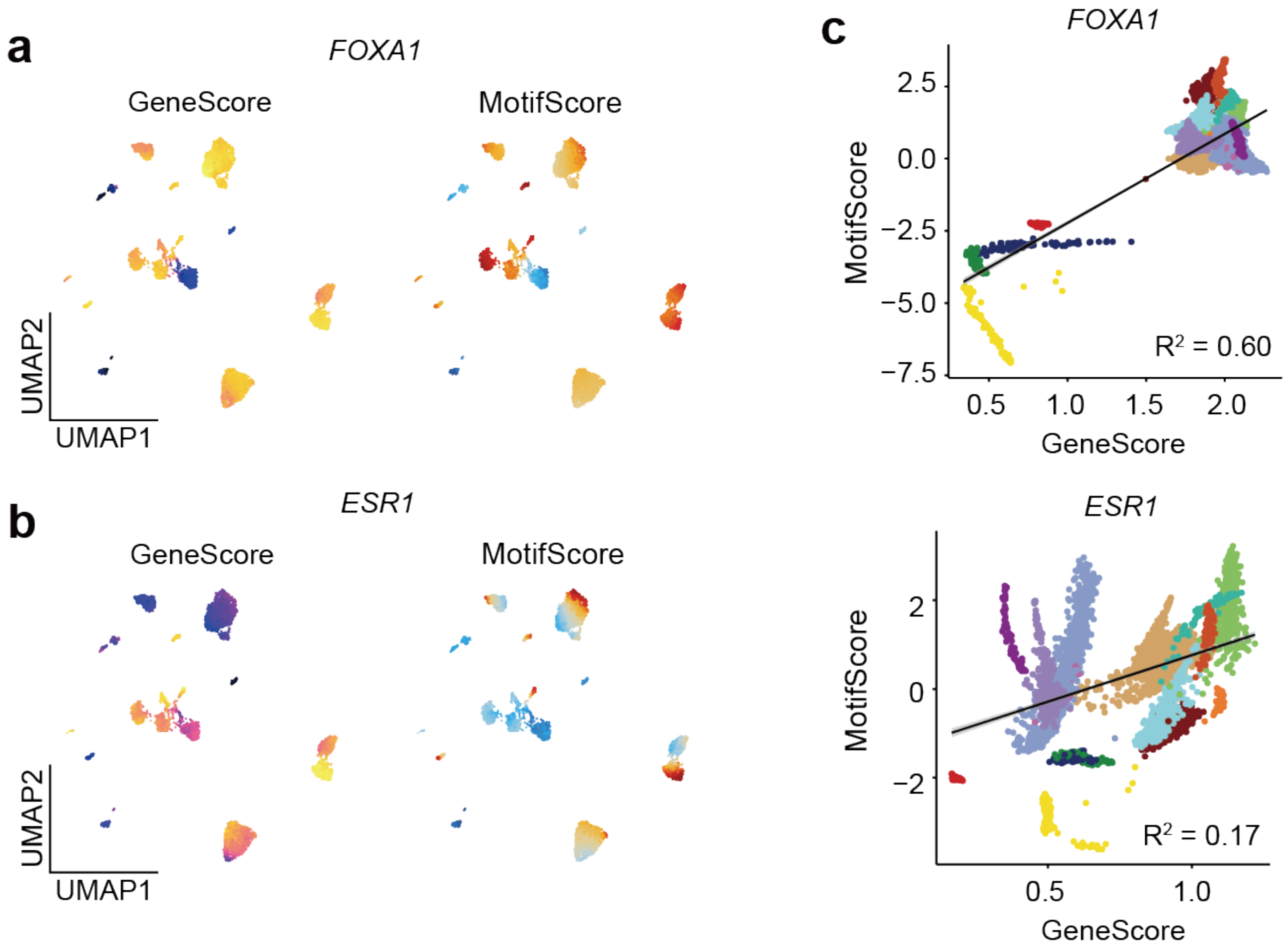


**Supplementary Figure 4. FOXA1 and ESR1 gene score and motif enrichment**

a, b UMAP overlay of gene activity and motif enrichment of FOXA1 in a, ESR1(ER) in b. c Scatter plot of gene activity and motif enrichment of FOXA1(top) and ESR1(bottom). Each dot represents each cell, colored by corresponding epithelial clusters in Figure 2a.

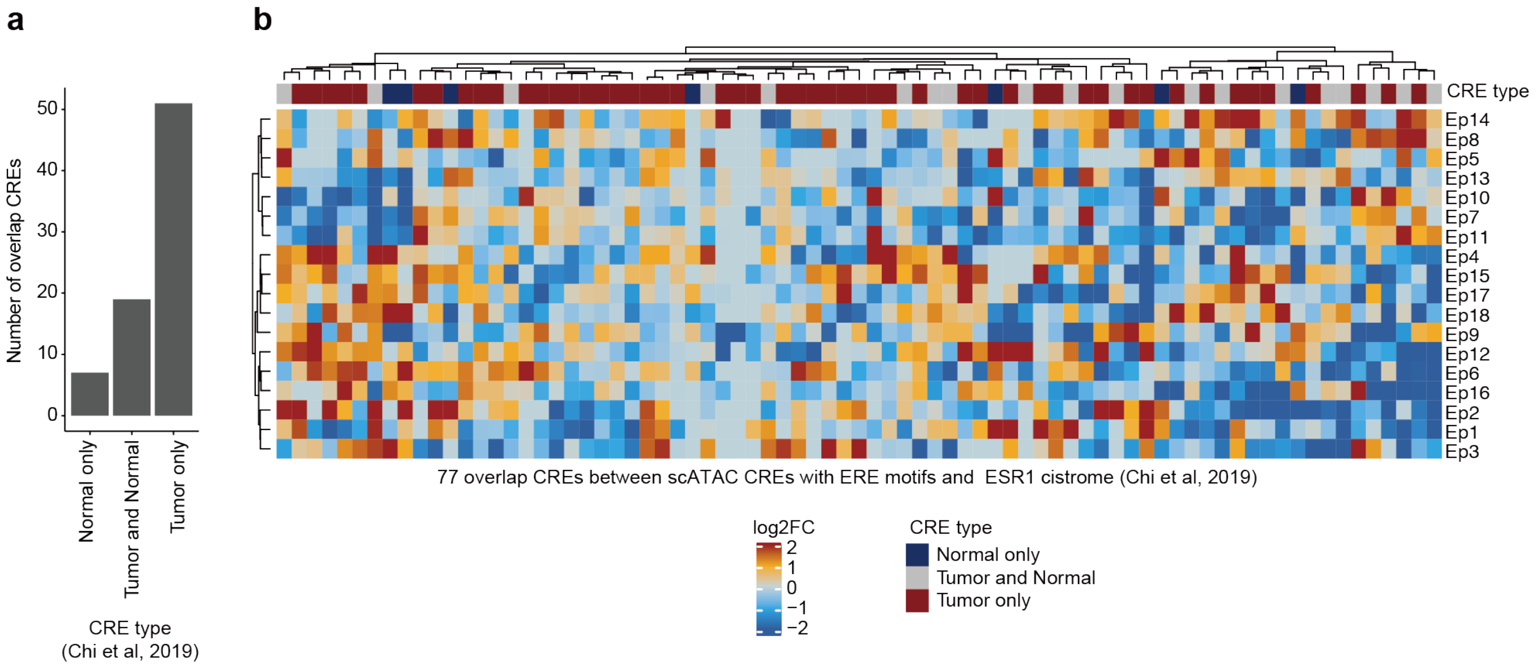


**Supplementary Figure 5. Overlap CREs between ER motif containing peaks identified by scATAC-seq and previously reported ER cistrome in primary breast cancer and normal epithelium**

a Numbers of overlap CREs between our scATAC-seq peak set of epithelial cells and ER bound sites in breast cancer cell and normal epithelium identified by Chi et al., 2019. b Heatmap of relative activity of 77 overlapped ER CREs.

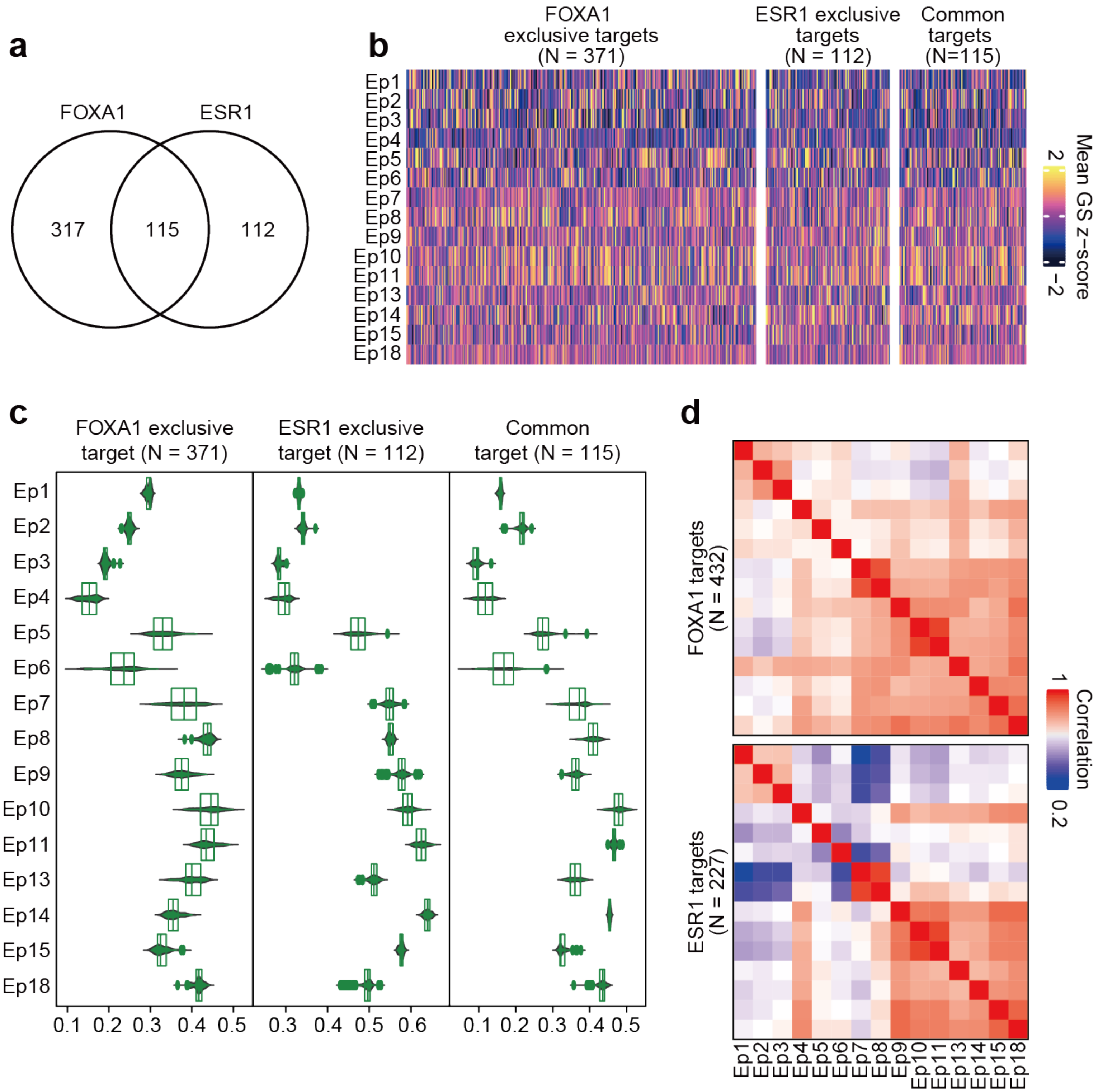


**Supplementary Figure 6. FOXA1 and ER target gene activity profiling**

a Venn diagram of the target genes of FOXA1 and ESR1 identified by Cistrome Cancer. b Heatmap of Z-scores of gene activity scores of FOXA1 exclusive, ER exclusive, and common target genes for each cancer cluster. c Signature scores of FOXA1 exclusive, ER exclusive, and common target genes for each cancer cluster. d Heatmap of Pearson’ s correlation of gene activity scores of FOXA1(top) and ER(bottom) targets.

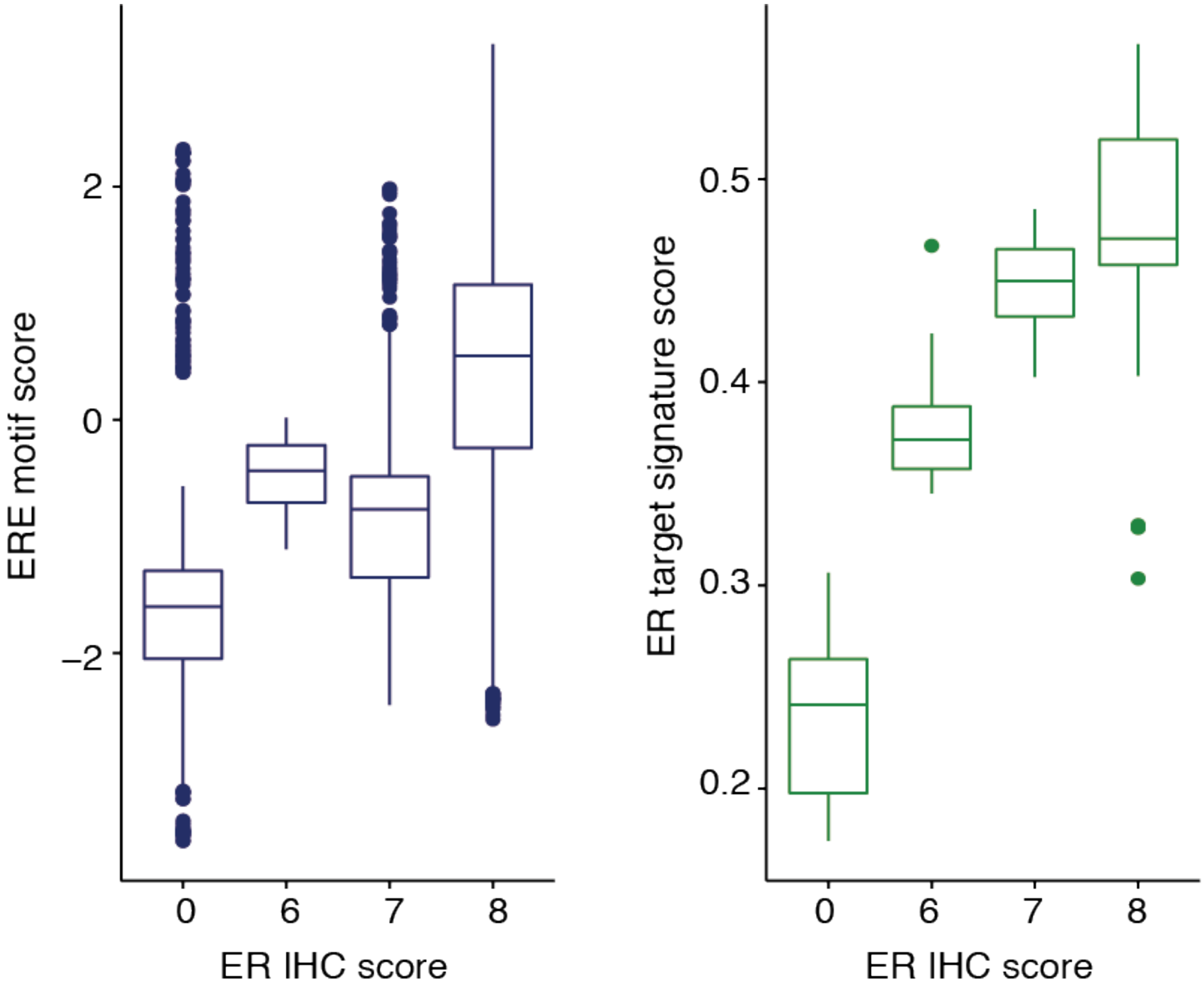


**Supplementary Figure 7. Comparison between ER IHC score and motif enrichment or signature score.**

Boxplots showing ERE motif enrichment score (left) or ER target signature score (right) stratified by ER IHC scores.

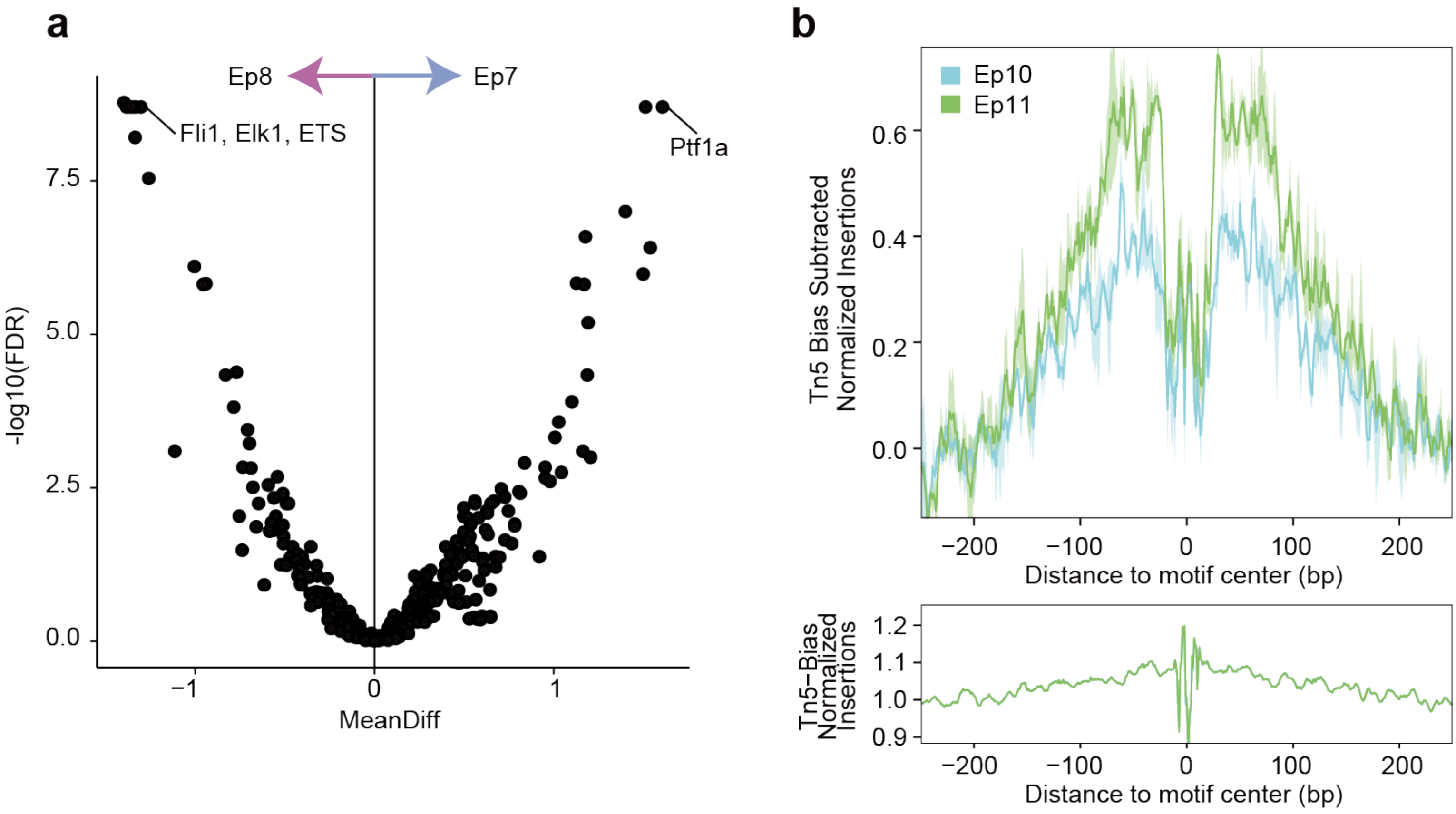


**Supplementary Figure 8. Intratumor heterogeneity of motif enrichment**

a Volcano plot representing differential motif enrichment between Ep7 and Ep8 from a single luminal tumor. b Tn5-adjusted transcription factor footprints for ER motifs in Ep10 and Ep11 clusters; Tn5 insertion tracks are presented below.
