# Supplementary material for: GRHL2 motif is associated with intratumor heterogeneity of cis-regulatory elements in luminal breast cancer
Source: NPJ Breast Cancer. 2022 Jun 8;8:70. doi: 10.1038/s41523-022-00438-6 (PMC9177858; doi:10.1038/s41523-022-00438-6)
Supplement: Supplementary file 2 — Reporting Summary [file 41523_2022_438_MOESM2_ESM.pdf]

nature portfolio

Corresponding author(s): Reo Maruyama

Last updated by author(s): Apr 13, 2022

Reporting Summary

Nature Portfolio wishes to improve the reproducibility of the work that we publish. This form provides structure for consistency and transparency in reporting. For further information on Nature Portfolio policies, see our Editorial Policies and the Editorial Policy Checklist.

Statistics

For all statistical analyses, confirm that the following items are present in the figure legend, table legend, main text, or Methods section.

n/a	Confirmed	
☐	☒	The exact sample size (*n*) for each experimental group/condition, given as a discrete number and unit of measurement
☐	☒	A statement on whether measurements were taken from distinct samples or whether the same sample was measured repeatedly
☐	☒	The statistical test(s) used AND whether they are one- or two-sided<br>*Only common tests should be described solely by name; describe more complex techniques in the Methods section.*
☒	☐	A description of all covariates tested
☐	☒	A description of any assumptions or corrections, such as tests of normality and adjustment for multiple comparisons
☐	☒	A full description of the statistical parameters including central tendency (e.g. means) or other basic estimates (e.g. regression coefficient) AND variation (e.g. standard deviation) or associated estimates of uncertainty (e.g. confidence intervals)
☐	☒	For null hypothesis testing, the test statistic (e.g. *F*, *t*, *r*) with confidence intervals, effect sizes, degrees of freedom and *P* value noted<br>*Give P values as exact values whenever suitable.*
☒	☐	For Bayesian analysis, information on the choice of priors and Markov chain Monte Carlo settings
☒	☐	For hierarchical and complex designs, identification of the appropriate level for tests and full reporting of outcomes
☐	☒	Estimates of effect sizes (e.g. Cohen's *d*, Pearson's *r*), indicating how they were calculated

Our web collection on statistics for biologists contains articles on many of the points above.

Software and code

Policy information about availability of computer code

Data collection	Bio-Rad ATAC-seq Analysis Toolkit – Barcode identification, alignment, filter, deduplication
Data analysis	R version 4.0.5 – R environment<br>ArchR 1.0.2 – Comprehensive analysis for scATAC-seq data<br>MACS2 2.2.7.1 – Peak calling<br>Seurat 4.0.5 – Comprehensive analysis for scRNA-seq<br>HOMER 4.10 – Motif enrichment analysis and differential peak detection

For manuscripts utilizing custom algorithms or software that are central to the research but not yet described in published literature, software must be made available to editors and reviewers. We strongly encourage code deposition in a community repository (e.g. GitHub). See the Nature Portfolio guidelines for submitting code & software for further information.

Data

Policy information about availability of data

All manuscripts must include a data availability statement. This statement should provide the following information, where applicable:

- Accession codes, unique identifiers, or web links for publicly available datasets
- A description of any restrictions on data availability
- For clinical datasets or third party data, please ensure that the statement adheres to our policy

scATAC-seq data are accessible through the NCBI Gene Expression Omnibus (GEO) with the accession number GSE198639.

Field-specific reporting

Please select the one below that is the best fit for your research. If you are not sure, read the appropriate sections before making your selection.

☒ Life sciences ☐ Behavioural & social sciences ☐ Ecological, evolutionary & environmental sciences

For a reference copy of the document with all sections, see nature.com/documents/nr-reporting-summary-flat.pdf

Life sciences study design

All studies must disclose on these points even when the disclosure is negative.

Sample size	scATAC-seq data were obtained from 16 breast cancer patients.
Data exclusions	No data were excluded from the study.
Replication	All measurements are not replicated.
Randomization	n/a. The data and analysis for this study is objective and not prone to influence by the researchers' bias.
Blinding	n/a. The data and analysis for this study is objective and not prone to influence by the researchers' bias.

Reporting for specific materials, systems and methods

We require information from authors about some types of materials, experimental systems and methods used in many studies. Here, indicate whether each material, system or method listed is relevant to your study. If you are not sure if a list item applies to your research, read the appropriate section before selecting a response.

Materials & experimental systems

n/a	Involved in the study	
☐	☒	Antibodies
☒	☐	Eukaryotic cell lines
☒	☐	Palaeontology and archaeology
☒	☐	Animals and other organisms
☐	☒	Human research participants
☒	☐	Clinical data
☒	☐	Dual use research of concern

Methods

n/a	Involved in the study	
☒	☐	ChIP-seq
☒	☐	Flow cytometry
☒	☐	MRI-based neuroimaging

Antibodies

Antibodies used	Estrogen receptor (Ventana,SP1,ready to use), Progesterone receptor (Ventana,1E2,ready to use), HER2 (Ventana,4B5,ready to use), Androgen receptor (Dako,AR441,1/200), FOXA1 (Abcam,EPR10881-14,1/1000)
Validation	All antibodies are commercially available and have validation statement from manufacturers for the application used.

Human research participants

Policy information about studies involving human research participants

Population characteristics	All patient samples were obtained by Japanese Foundation for Cancer Research in accordance with the Declaration of Helsinki after written consent from the patients upon enrolling in the trial.
Recruitment	Patients did not receive compensation for participation in this study.
Ethics oversight	Informed consent was obtained from donors, and the study was approved by Institutional Review Board at the Japanese Foundation for Cancer Research under license No.2018-1168.

Note that full information on the approval of the study protocol must also be provided in the manuscript.
